# Performance of non-parametric algorithms for spatial mapping of tropical forest structure

**DOI:** 10.1186/s13021-016-0062-9

**Published:** 2016-08-24

**Authors:** Liang Xu, Sassan S. Saatchi, Yan Yang, Yifan Yu, Lee White

**Affiliations:** 1 Institute of the Environment and Sustainability, University of California, Los Angeles, CA 90095 USA; 2 Jet Propulsion Laboratory, California Institute of Technology, Pasadena, CA 91109 USA; 3 Department of Earth and Environment, Boston University, Boston, MA 02215 USA; 4 Agence National des Parks Nationaux, Battery 4, B.P. 20379, Libreville, Gabon

**Keywords:** Tropical forests, Canopy height, Lidar, Spatial mapping, Gabon

## Abstract

**Background:**

Mapping tropical forest structure is a critical requirement for accurate estimation of emissions and removals from land use activities. With the availability of a wide range of remote sensing imagery of vegetation characteristics from space, development of finer resolution and more accurate maps has advanced in recent years. However, the mapping accuracy relies heavily on the quality of input layers, the algorithm chosen, and the size and quality of inventory samples for calibration and validation.

**Results:**

By using airborne lidar data as the “truth” and focusing on the mean canopy height (MCH) as a key structural parameter, we test two commonly-used non-parametric techniques of maximum entropy (ME) and random forest (RF) for developing maps over a study site in Central Gabon. Results of mapping show that both approaches have improved accuracy with more input layers in mapping canopy height at 100 m (1-ha) pixels. The bias-corrected spatial models further improve estimates for small and large trees across the tails of height distributions with a trade-off in increasing overall mean squared error that can be readily compensated by increasing the sample size.

**Conclusions:**

A significant improvement in tropical forest mapping can be achieved by weighting the number of inventory samples against the choice of image layers and the non-parametric algorithms. Without future satellite observations with better sensitivity to forest biomass, the maps based on existing data will remain slightly biased towards the mean of the distribution and under and over estimating the upper and lower tails of the distribution.

## Background

Structure and aboveground biomass (AGB) of tropical forests are highly variable, creating landscape scale heterogeneity that cannot be readily captured without spatial mapping or increasingly dense systematic sampling [1–3]. The uncertainty in the distribution of AGB and forest structure at local to regional scales has impacted the estimation of greenhouse gas (GHG) emissions and removals from land use activities [4, 5], the monitoring of the global carbon cycle [6], and the development of climate change mitigation policies in the framework of REDD (reduced emissions from deforestation and degradation) [7]. Local and landscape heterogeneity of tropical forest biomass and structure are larger than regional and continental scale variations [1, 8–11], making extrapolation or estimation of finer scale variations difficult and uncertain [2].

Increasing availability of airborne observations of tropical forests with Lidar and radar remote sensing techniques, has significantly improved our ability to quantify and map forest structure at higher spatial resolution [12–15]. Unlike conventional passive optical sensors, these active sensors can capture the vertical vegetation structure by either measuring the range of laser light reflected from vegetation elements and the ground [16, 17], or measuring the radar backscatter and phase at a given wavelength and polarization [15, 18–20]. Among these new techniques, high resolution (small footprint) airborne Lidar data can provide the most accurate measurements of forest height and vertical structure, allowing estimation of forest biomass with reasonably low uncertainty compared to ground estimates [12, 21, 22]. However, in the absence of spatial measurement of forest structure at larger scales, often samples of ground or Lidar data are used to develop maps using non-parametric or machine-learning techniques with the aid of active and passive satellite imagery or environmental variables [15, 23]. The uncertainty of the maps depends strongly on the non-parametric algorithm that is used as an estimator of forest structure for each mapping unit or pixel, the quality of satellite imagery and the environmental variables, and the quality and quantity of samples used to train the non-parametric models [24].

The Lidar-derived metric, mean canopy height (MCH), or the centroid of the vertical canopy profile [25], has been found to be the best variable calibrating AGB as a single-metric model [21, 26]. Although MCH may underestimate the canopy heights in complex terrain under the circumstance of reduced data density [27], it has excellent agreements with field-measured maximum and mean tree heights at least in low-slope environments [28] as well as the field-measured basal area [29]. However, airborne Lidar still exists as a sampling tool in most regions of the tropics, because the wall-to-wall Lidar data are scarce. For large-scale mapping, airborne/satellite Lidar is sampled as a surrogate for tropical forest structure and use radar and optical imagery for spatial modeling and mapping [12, 15, 30–32]. In mapping forest structure at medium-to-high resolution (e.g. 100-m), high-quality satellite data include the Landsat series in the optical domain, the L-band radar backscatter data collected from the Advanced Land Observing Satellite “DAICHI” (ALOS) platform and the digital elevation model (DEM) data derived from the Shuttle Radar Topography Mission (SRTM) in the microwave domain. The accuracy of the maps depends strongly on the quality and relevance of satellite input layers, the model setup suitable for tropical forest structure prediction, as well as the design and selection of the training samples.

**Fig. 1:**
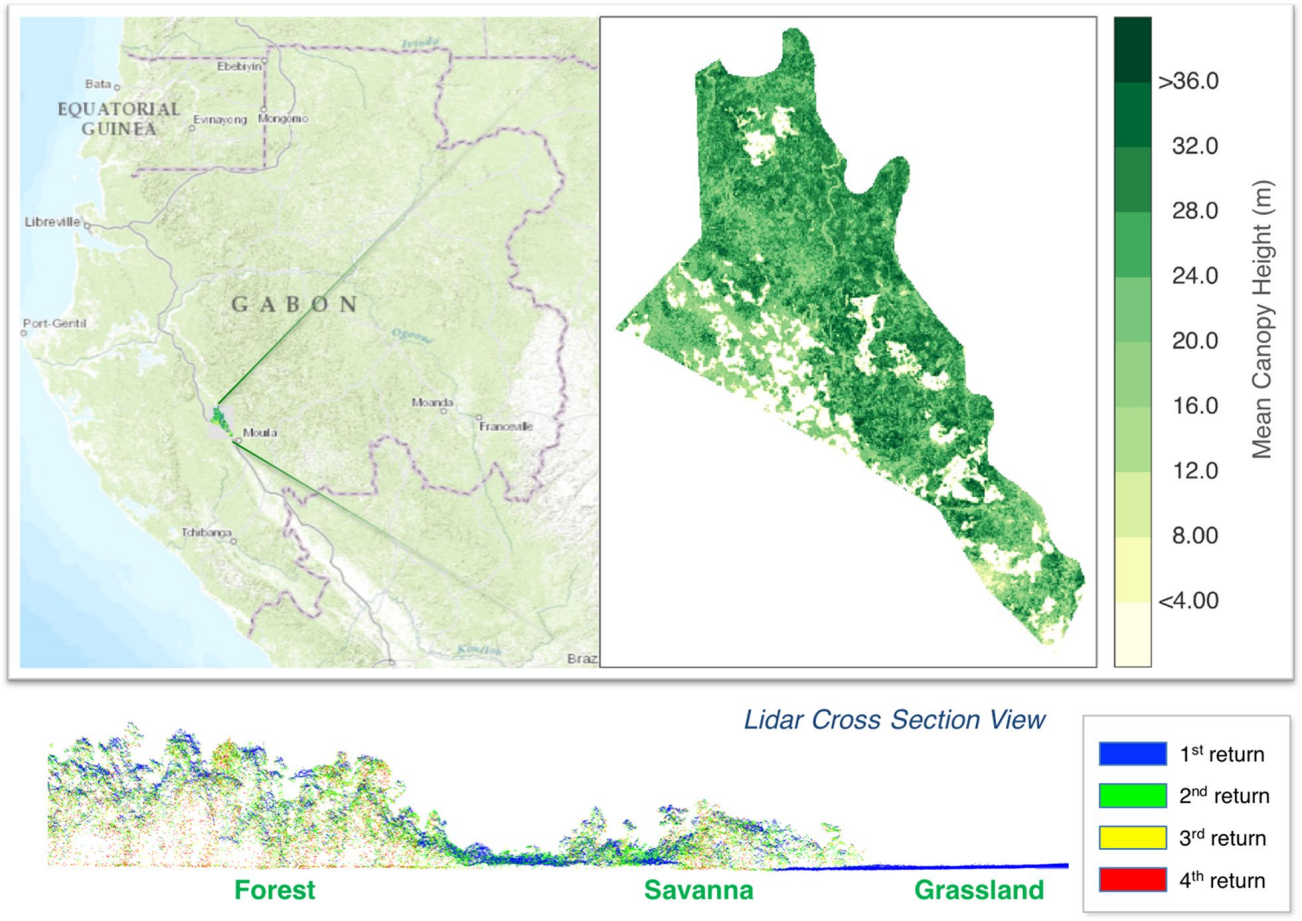
Study site in Mouila, Gabon. The mean canopy height (MCH) was spatially averaged to 100 × 100 m from airborne lidar-derived CHM product at 1-m spatial resolution

In this study, we utilize small footprint airborne lidar data to evaluate the performance of high-resolution mapping of tropical forest structure from satellite layers using popular machine learning algorithms. The study area is located north of the City of Mouila in Ngounie province in southwestern Gabon, covering approximately 33,500 ha of moist tropical forests and savanna west of Ngounie river. Gabon is the second most forested country in the world after Surinam, with 85 % (24,000 km^2^) of its area (267,667 km^2^) covered by tropical rainforest [15]. The area selected for this study is relatively flat with approximately 40 m variations in elevation from 80 to 120 m above the sea level. The forests near the flat savanna areas are seasonally inundated, increasing the heterogeneity of the forest structure in the study area. The structure of vegetation varies from savanna, grass and shrublands to successional forests near the forest-savanna boundaries, degraded forests near villages, and extensive intact old growth upland forests away from the savanna vegetation and slightly on higher elevation. The Mouila site was selected to extend the palm oil plantations in sustainable production [33], where the whole site was measured by airborne lidar for carbon assessment (Fig. 1).

Given the fact that Lidar-derived MCH can act as the ground (airborne) truth representing forest structure and carbon stocks with only a few field plots to stabilize [21, 23], we focus on MCH mapping from satellite data using a small number of training samples. Through the use of non-parametric models, the random forest (RF) and the Maximum Entropy (ME) algorithms, we try to answer the following questions: (1) What types of information should be included as input layers to improve the model predictions? (2) How can we adjust the learning algorithms to suit our needs for unbiased estimations, especially for large trees? (3) What is the effectiveness of forest structure mapping from current satellite data using a limited quantity of ground measurements? For regional-scale analyses, sampling strategy is one of the key components for successful mapping of forest structure. The continuous coverage of Lidar-derived MCH gives us the opportunity to showcase a set of benchmark tests of satellite retrievals for forest structure and carbon stocks using empirical (machine learning) methods. We expect these results can serve as guidelines for parameter tunings of machine learning algorithms dedicated to tropical forest structure retrievals, and also help to better design future field plots at local to regional scales.

## Results and discussion

### Passive optical, radar backscatter, interferometry, and texture information

**Fig. 2:**
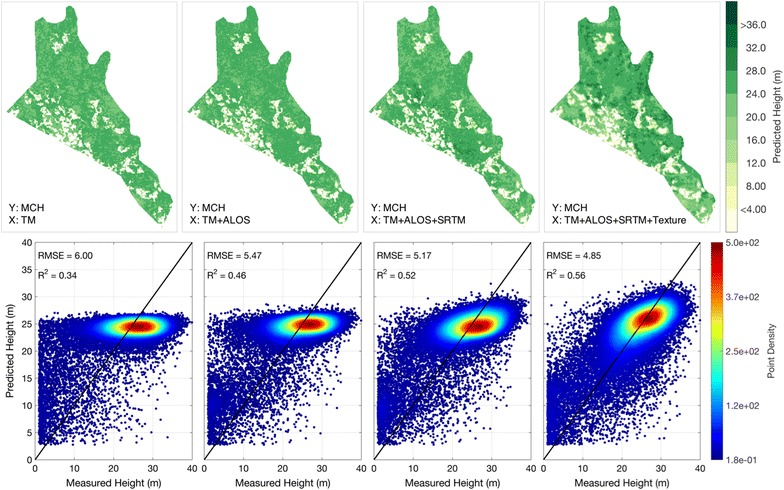
Mapping results of ME using different input layers. The *upper panels* show ME prediction maps trained from 400 randomly selected samples of tropical forest MCH. The *lower panels* show scatter plots of test samples that are not included in training

**Fig. 3:**
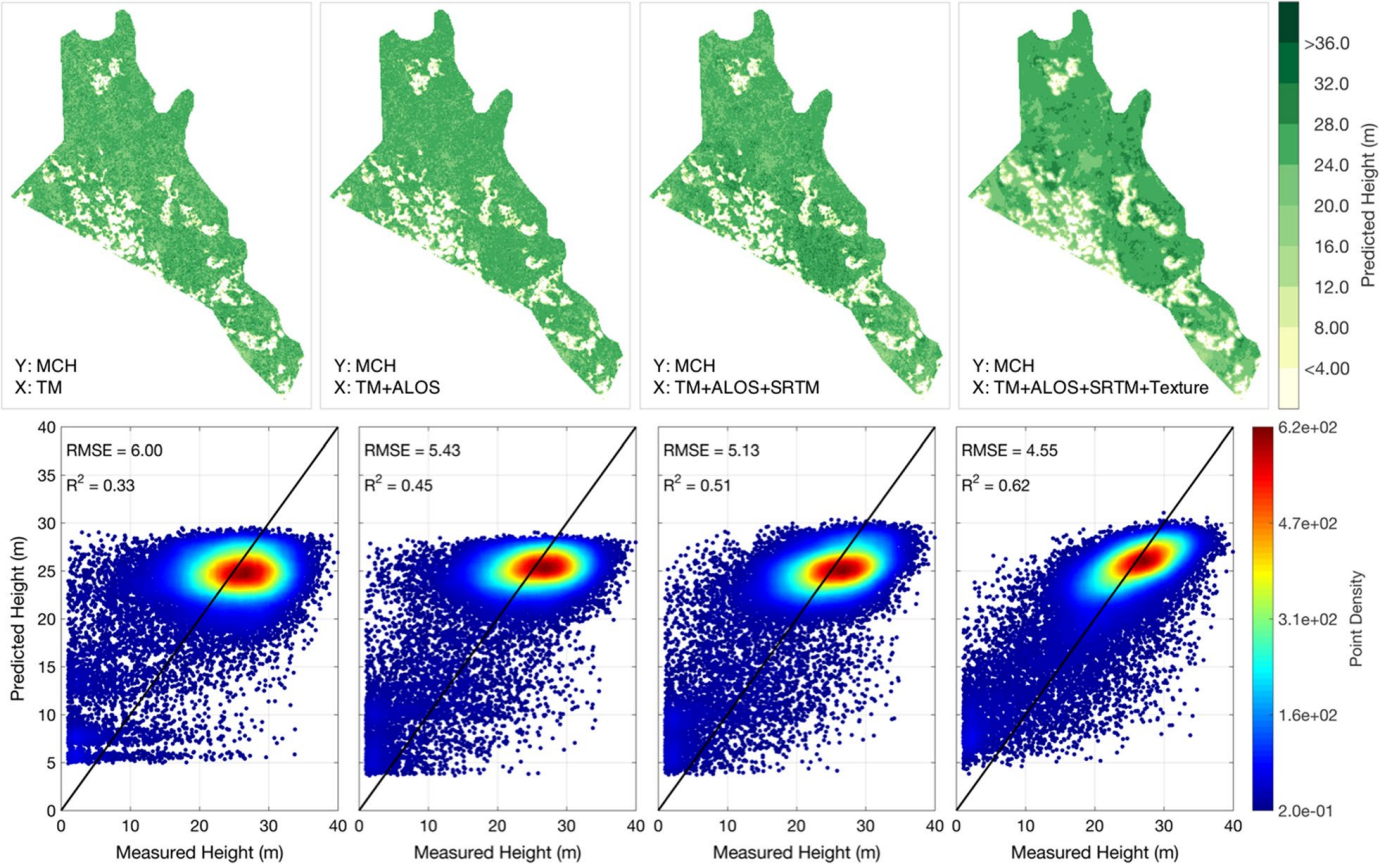
Similar mapping results as Fig. 2, but from RF models

Using default settings of both RF and ME algorithms, we tested the MCH prediction capability of Landsat, ALOS and SRTM in 100-m spatial resolution, and found improved predictions of MCH when adding more information from different satellite layers (Figs. 2, 3). Both algorithms produce similar prediction accuracy regarding root-mean-square error (RMSE) or coefficient of determination (R^2^). By conducting Monte Carlo cross validations (CV), the RMSE of RF method decreases from 6.02 ± 0.10 m (predicted from Landsat data alone) to 5.06 ± 0.07 m when using all inputs from three satellite sensors (Table 1). R^2^ values have an improvement from 0.33 ± 0.02 to 0.53 ± 0.01. ME results have a comparable RMSE (R^2^) accuracy, starting from 6.00 ± 0.10 m (R^2^ = 0.34 ± 0.02) for Landsat only predictions, to 5.17 ± 0.09 m (R^2^ = 0.52 ± 0.01) for predictions from all three sensors. Tests using data from each satellite sensor show similar prediction accuracies (Table 1), with ALOS being the most sensitive to MCH, but none of them has the comparative prediction power of all sensors combined. The significant improvements with the addition of layers suggest that the data fusion from different sensors can help to achieve better prediction results.

**Table 1:** Machine learning performance using different input layers

Input layers	L only	A only	S only	L + A	L + A + S	L + A + S + T	BC
RF							
RMSE	6.02 ± 0.10	5.80 ± 0.08	6.20 ± 0.04	5.41 ± 0.07	5.06 ± 0.07	4.51 ± 0.07	4.58 ± 0.09
R^2^	0.33 ± 0.02	0.39 ± 0.02	0.30 ± 0.01	0.46 ± 0.01	0.53 ± 0.01	0.63 ± 0.01	0.63 ± 0.01
MSD	0.08 ± 0.23	−0.13 ± 0.33	−0.16 ± 0.31	0.10 ± 0.22	0.12 ± 0.22	0.08 ± 0.25	−0.08 ± 0.18
MSD1	3.68 ± 1.20	2.32 ± 1.15	6.84 ± 1.25	3.80 ± 1.21	3.89 ± 1.24	4.48 ± 0.99	0.71 ± 0.81
MSD2	−8.50 ± 0.45	−7.63 ± 0.60	−7.03 ± 0.81	−7.46 ± 0.50	−5.61 ± 0.59	−5.16 ± 0.43	−1.73 ± 0.57
ME							
RMSE	6.00 ± 0.10	5.62 ± 0.06	6.12 ± 0.05	5.46 ± 0.09	5.17 ± 0.09	4.73 ± 0.13	5.29 ± 0.17
R^2^	0.34 ± 0.02	0.42 ± 0.01	0.32 ± 0.01	0.46 ± 0.02	0.52 ± 0.01	0.59 ± 0.02	0.53 ± 0.02
MSD	0.10 ± 0.23	−0.12 ± 0.31	−0.16 ± 0.34	0.10 ± 0.24	0.05 ± 0.25	0.02 ± 0.24	−0.15 ± 0.17
MSD1	5.76 ± 0.71	4.27 ± 1.04	9.00 ± 1.33	4.71 ± 0.67	4.71 ± 0.56	4.57 ± 0.38	1.10 ± 0.64
MSD2	−10.88 ± 0.50	−9.88 ± 0.69	−8.24 ± 1.18	−8.78 ± 0.90	−6.73 ± 0.73	−4.39 ± 0.24	−1.13 ± 0.34

The sample size for this test was fixed at 400 samples, and the rest 32,674 100-m pixels were used as test samples. The results were cross validated by repeated random sampling of the training data (Monte Carlo CV). RF and ME predictions were evaluated using RMSE, R^2^, overall MSD, MSD for small trees (MSD1) and the MSD for large trees (MSD2). The input “L only” includes four Landsat bands, “A only” uses only the two ALOS bands, “S only” uses only the SRTM bands, “L + A” includes four Landsat and two ALOS bands, “L + A + S” includes Landsat, ALOS and SRTM bands, “L + A + S + T” includes all satellite bands plus texture layers, and “BC” uses the same set of input layers as “L + A + S + T”, but results are from the bias-corrected algorithms

However, neither model shows an unbiased prediction along the one-to-one line of the actual MCH values. The scatter plots of test data (lower panels in Figs. 2, 3) show a vast majority of predictions around 25 m, thus having a flattened oval shape along the X-axis. This pattern of deviation from the one-to-one line means an underestimation of high MCH and an overestimation of low MCH. Although the overall mean signed deviation (MSD) is small when calculated from all test points, the MSD for large trees (MSD2) reveals a significant underestimation that is consistently lower than the measured MCH by 5–10 m. But this quantity also improves with increased number of input layers, meaning the extra information provided by additional layers increases the sensitivity of input signal to large trees. Similarly, the MSD for small trees (MSD1) has an overestimation of 4–6 m, and it does not improve much with more input layers.

In addition to the original input bands from satellite observations at corresponding locations, we also tested the contribution of surrounding pixels to the MCH prediction. Both RF and ME prediction results show a further improvement when adding texture layers (last column of Figs. 2, 3). The texture information brings an additional half meter decrease in RMSE for the RF results (0.44-m decrease for ME), and improves the R^2^ by 0.1 (0.07 for ME) (Table 1). Another important contribution of adding texture is making the predictions less biased. There are much more pixels with predicted MCH over 30 m, compared to the predictions without texture. The red oval, representing the majority of test data in the scatter plot (Figs. 2, 3), also reclines more toward the one-to-one line of MCH. Although it does not change a lot on the overall MSD, which is always unbiased, the MSD2 appears to be less biased (−5.16 m for RF, and −4.39 for ME).

### Model adjustments dedicated to tropical forests

**Fig. 4:**
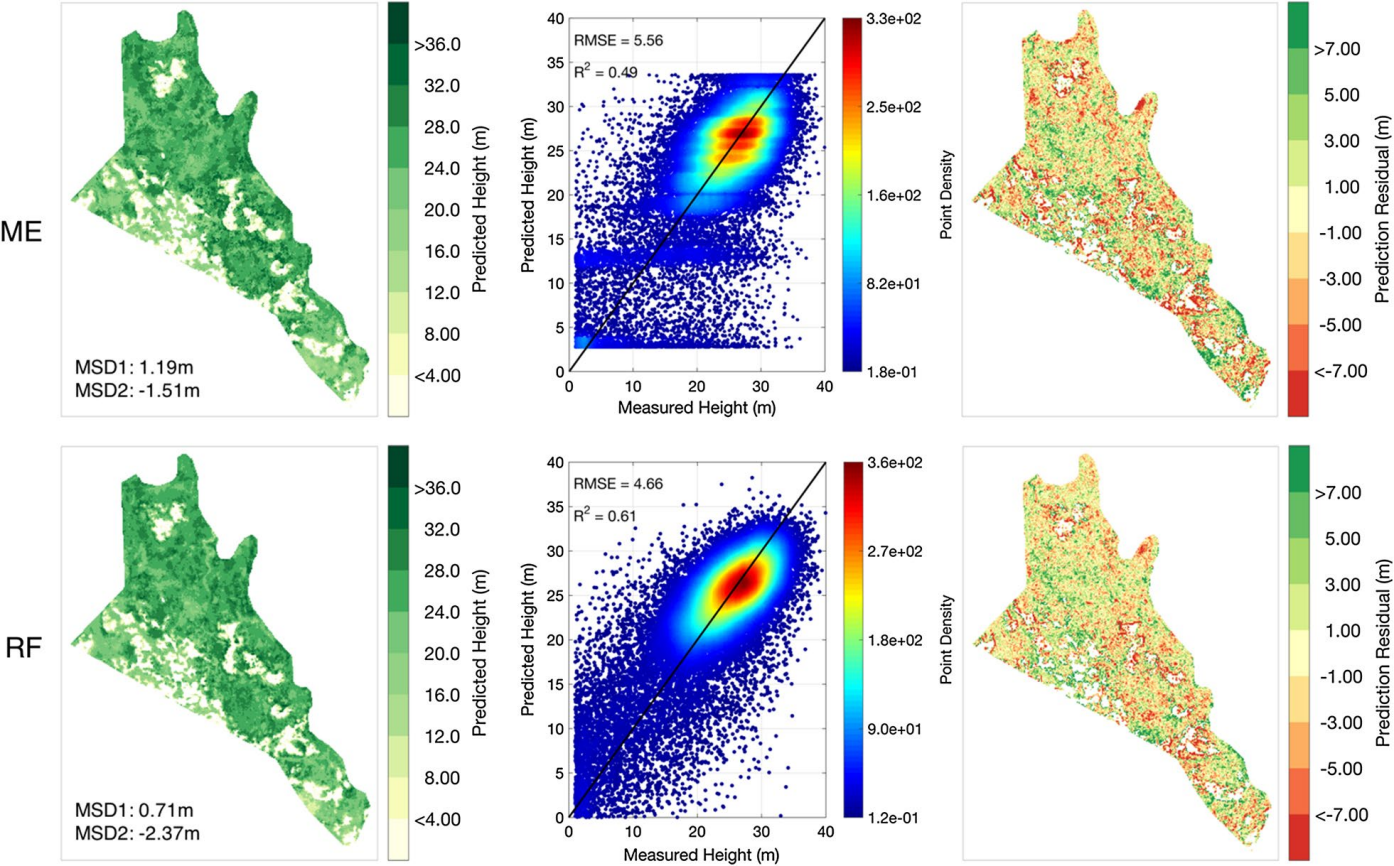
Bias-corrected results of ME (*upper panels*) and RF (*lower panels*) models. The *first column* shows the prediction maps of MCH, the *second column* shows the scatter plots of test samples, and the *third column* shows residual maps when comparing to the measured MCH

Unbiased estimation of large trees is important in the structure retrieval of tropical forests, as they are the dominant component in biomass and carbon stocks. Bias correction is, therefore, the major task of the model adjustment in tropical forests. Results of ME adopting the published tuning method [15, 34] will reduce the bias at the cost of more dispersed predictions, i.e., larger RMSE and smaller R^2^ values (Fig. 4). Using the same settings of training and test samples, the bias-corrected ME (MEBC) shows significant reductions in MSD1 from 4.57 to 1.10 m, and MSD2 from −4.39 to −1.13 m (Table 1). However, MEBC loses accuracies in RMSE and R^2^ (RMSE from 4.7 to 5.3, and R^2^ from 0.59 to 0.53) as an adverse effect of bias correction.

The application of the bias correction method on the RF results in similar prediction accuracy as the original RF with a significant improvement in MSD1 and MSD2. The Monte Carlo CV results also indicate that this improvement is not a coincidence from a single realization (Table 1). The bias-corrected version of RF (RFBC) has comparable R^2^ as the original RF (0.63 ± 0.01), a slightly larger RMSE (4.60 m), and a significantly reduced MSD1 from 4.5 to 0.7 m, and MSD2 from −5.4 to −1.7 m.

### Effects of sample size

It is well known that increasing the training sample size can help to improve the prediction accuracies, but it is unclear whether the improvements in MSD using the bias-corrected methods can also be achieved by increasing the sample size using original methods. Alternatively, it is important to test whether the sample size increase can compensate the losses in RMSE and R^2^ when using bias-corrected methods.

**Fig. 5:**
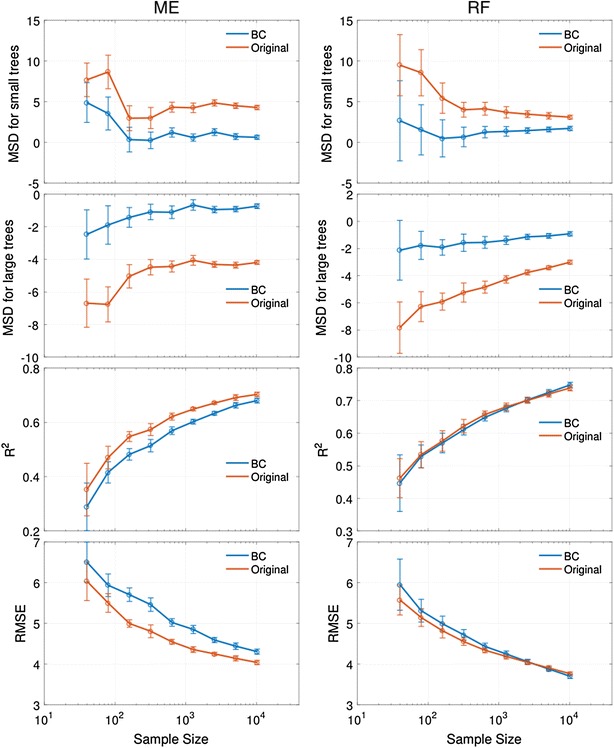
Statistical measures of ME (*left panels*) and RF (*right panels*) performance with various sample sizes. We tested MSD1 (*first row*), MSD2 (*second row*), R^2^ (*third row*) and RMSE (*fourth row*). The test sample size was fixed at 5000 for all tests when varying the size for training samples. The tests mainly compared the measures from the original (Original) and the bias-corrected version (BC) of the models

Monte Carlo CV tests (Fig. 5) comparing RF and RFBC show that for sample size up to ten thousand (around one-third of all valid observations), the original RF can never achieve the same level of MSD2 (around −2 m) by using RFBC, even when RFBC has only 40 training samples. MSD1 from RFBC also shows consistently lower biases than what we get from the original RF model. Meanwhile, RF and RFBC results have very similar R^2^ values across all sample sizes, and RFBC has a slightly larger RMSE than the original RF when the sample size is below 2500. The result suggests that predictions using RFBC can always achieve the same level of accuracy by increasing the sample size. For example, on average there is a 0.2-m difference in RMSE between using RF and using RFBC when we have 80 training samples, but this lower RMSE obtained from RFBC can be easily improved by adding around 30–40 training samples. A more interesting finding of RFBC is that the accuracy exceeds the original RF in all statistical metrics when the sample size is large enough (greater than 2500), suggesting that our proposed bias correction of RF is an overall better model compared to the original for large sample size.

ME results also provide better predictions in MSD1 and MSD2 (Fig. 5), but regarding R^2^ or RMSE, MEBC approach needs more training samples to achieve the same accuracy of ME. Using the same example of the sample size of 80, a half-meter difference in RMSE between ME and MEBC predictions requires about 200 additional training samples to amend the RMSE loss. This is practically not cost-effective when considering all the efforts required for the ground data acquisitions and logistics in tropical forests.

### Spatial autocorrelation

**Fig. 6:**
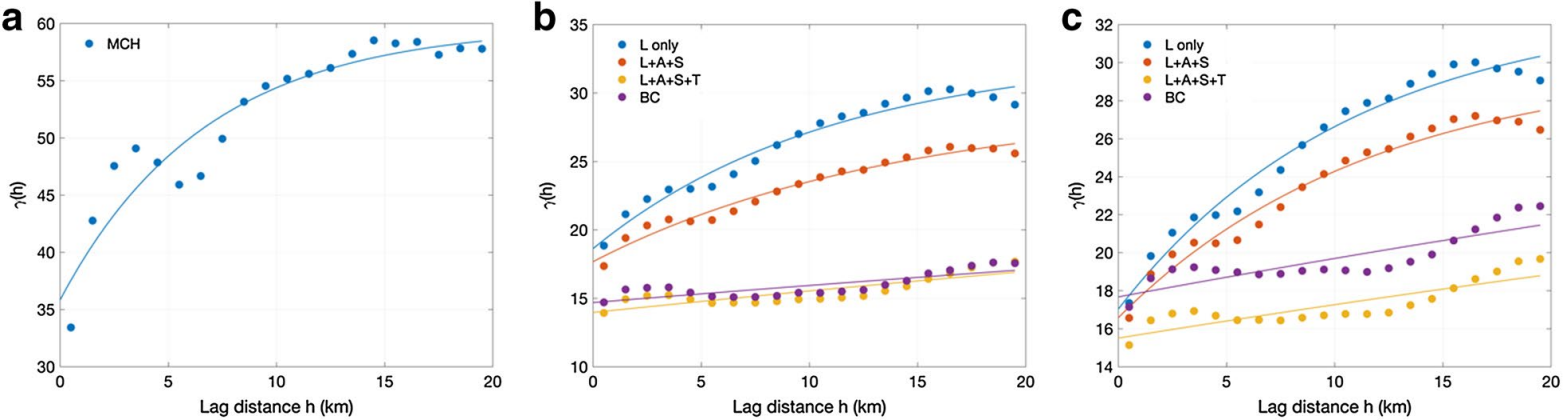
Semi-Variogram plots of **a** the Lidar-derived MCH map, **b** the residual map from different RF models, and **c** the residual map from different ME models. Naming of the legend items can be found in Table 1

Spatial autocorrelation exists when the value of a pixel is predictable from nearby pixels. Although tropical forests are usually dense and heterogeneous, spatial patterns are not completely random, and the variogram plot of measured MCH confirms spatial dependence on horizontal distance (Fig. 6a). Previous studies have shown that estimations from sampling data without considering spatial information could introduce large biases and erroneous spatial representations [1, 2], and thus systematic sampling from remote sensing data is crucial to the accurate mapping of tropical forests at the regional scale. We also found that the geolocation information aids to improve the remote sensing based predictions substantially, assuming that spatial autocorrelations can explain the model residuals. Spatial autocorrelation information can be either modeled through the variance–covariance structure of regression residual in Kriging methods [35] or directly included as another set of predictor variables [24].

In our study, we assumed that the information relating to spatial context could be estimated from surrounding satellite data or texture information, due to the existence of spatial autocorrelation [3, 9]. Texture derived from satellite data is important for high-resolution mapping as it brings additional spatial information for estimating the forest structure from the knowledge of surrounding pixels. The model prediction improvements were significant when adding texture layers (e.g. Table 1; Figs. 2, 3). From the variogram plots (Fig. 6b, c) showing the variance along the paired distance, we also found the flattened slope of semi-variogram for predictions with texture information, suggesting that a large part of spatial dependence at the short distance were explained by the texture layers included in the model. The variogram slopes derived from BC model residuals do not differ much from the original models, indicating that the algorithm tuning for bias correction has no significant impact on the spatial autocorrelation.

### Comparison of model performance

The performance of original spatial models is approximately the same on the average. However, when they are tuned to predict the height of tropical forests over the entire range, there are differences when comparing the results on pixel-by-pixels. These differences include (1) a slightly higher bias in RF results towards the mean of samples, reducing the variability in pixels at the tails of the distribution compared to ME. After the bias correction, the difference between RF and ME results still show slight overestimations of RF model in areas with low values and underestimation in areas of high values (e.g. see MSD1 and MSD2 values in Fig. 5). This is due to the differences between RF and ME algorithms. In MEBC, we simply assign more weights on the most probable classes for correcting biases [15, 34], while in RFBC, we build a second RF model to estimate the biases generated from the original RF model. (2) RF provides much higher prediction accuracy over pixels used for training data compared to the test data, whereas the ME predictions showed similar accuracy on both training and test data, suggesting the high dependence on training data in RF models. Although the RF methodology is designed to avoid overfitting [36], there are indications that it tends to capture mistakes in training for small sample size [37]. In contrast, ME methodology avoids overfitting by relying on a regularization technique and a Bayesian estimator that includes the prior probability distributions of both training and predictions. (3) For implementation, both techniques have similar computing time and will benefit from parallel processing when used with high-resolution data and multiple layers. Unlike the random forest, the maximum entropy model is designed to work with sub-optimum sample size as it predicts the distribution when it is under-sampled as in most signal processing problems [38]. When the sample size is large compared to the background layers, the ME methodology may produce additional uncertainty from the oversampling. This problem may not occur in random forest. This feature also suggests that the ME algorithm may be more suitable in cases where the number of ground samples is limited and small compared to the overall size of the mapping area.

### Residual bias

**Fig. 7:**
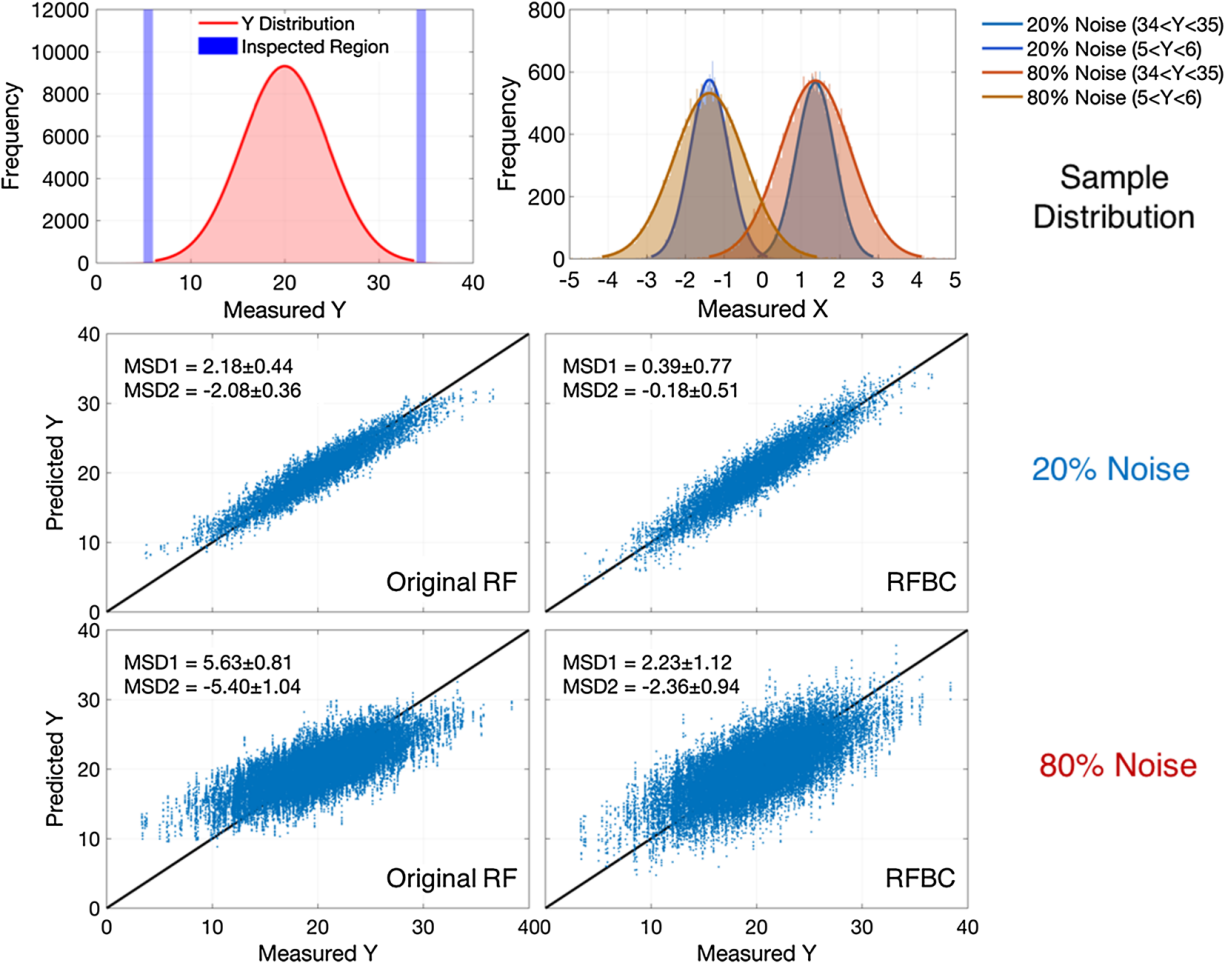
Model performance on the simulation data. The simulation data has two sets of independent variables (X)—with either 20 % noise or 80 % noise over the original X distribution. Sample distribution curves show two extreme examples of X distribution with large Y (34 < Y < 35, representing large trees) and low Y (5 < Y < 6, representing small trees). Original RF and RFBC were performed on these two sets of simulation data with half of the data as training and the rest as the independent test set

The bias-corrected models of RF and ME both show significant improvements regarding MSDs for small and large trees. However, small biases remain in the prediction residuals even when the sample size is large enough. For example, we still have MSD1 of around 1 m and MSD2 of −1 m when the RFBC model is trained using 5000 samples. Tests using simulated data with random noise generated in both predictor and response variables show that RFBC has an unbiased estimation for both MSD1 and MSD 2 with a slightly larger dispersion (RMSE or R^2^) than the original RF (20 % noise simulations in Fig. 7). If we generate a simulated dataset with larger noise, i.e., losing sensitivity to the response variable (80 % noise simulations in Fig. 7), both RF and RFBC have less accurate predictions as expected, and importantly, the proposed RFBC method cannot restore the unbiased predictions at both ends. Therefore, the remaining biases could come from sources of errors that are comparable to the signal itself, and thus hard to model or correct. This is particularly true in the case of tropical forests, as both radar and optical observations lose their sensitivity to dense forests and high biomass. The signal-to-noise ratio is very low in these types of forests, causing more estimations towards the mean value. The source of noise could be the effect of environmental conditions in the case of radar data, resulting in high backscatter in low density forests due to the effect of moisture level. The noise in optical data could come from the variations of leaf-level albedo and orientation that significantly alters the canopy-level reflectance, despite the strong impact from canopy structure. Additional effects of multiple scattering events and speckle noise within and between pixels can further blur the signal of forest structure contained in the satellite data.

## Conclusions

Using the tropical forest site in Mouila, Gabon as a case study, we explored the performance of spatial modeling under various circumstances. In particular, we investigated the structure retrieval capability using existing satellite imagery with limited sensitivity to forest structure variations. Using MCH as the variable of interest and airborne lidar measurements as the surrogate truth, we found: (1) The quality of retrieval improves with more information brought by additional satellite measurements and spatial texture layers. (2) The model adjustment for bias correction significantly improves the predictions for two edges (small and large trees). And (3) the loss of accuracy in RMSE or R^2^ due to bias correction algorithms can potentially be compensated by increasing the sample size of the training data.

With the current level of information and measurement errors obtained from remote sensing data, a certain amount of uncertainty in the final product of vegetation mapping is unavoidable, but our study shows that the mapping process can be optimized by considering the choice of inputs, the algorithms to use and the size of samples. With a focus on remote sensing data used in mapping tropical forest structure, we expect this study will help to utilize the existing machine learning algorithms better and modify them appropriately to suit the needs for tropical forest structure retrievals.

## Methods

### Remote sensing data

#### Airborne small-footprint lidar

The airborne small footprint Lidar data were acquired in September 2011 by SEPRET, a Moroccan company, using A Leica ALS 60 sensor mounted on a Cessna 402 airplane. The measurements were collected at a scan 96.1 KHZ, providing an average of 5–20 returns per m^2^. The data were processed at UCLA using a combination of TerraScan (version 013.021) and the FUSION Lidar processing software developed by the US Forest Service. The processing steps included the ground and top canopy classification to generate the wall-to-wall digital surface (DSM), terrain models (DTM), and consequently a canopy height model (CHM) from their difference posted at 1 m^2^ resolution over more than 33,500 ha of the Mouila complex. The data sets collected by SEPRET had an average of six points per m^2^ and provided on the average more than 5 % returns from ground that were classified and interpolated to create a seamless DTM over the entire study area. The Lidar data captures a variety of vegetation from savanna grasslands to transitional forest-savanna vegetation to dense, moist tropical forests. From the CHM data, we developed aggregated height by averaging the top canopy height of each pixel to generate mean canopy height (MCH) at 100-m (1-ha) pixels.

#### Satellite imagery

We used all four Landsat bands from the global mosaics of the Global Forest Change (GFC) research as the first input to the spatial modeling approach [39]. The images were from the 2012 Landsat cloud-free image composite (hereon referred to as “GFC Last”) is the multispectral imagery taken from the Landsat 7 satellite within the range of 1999–2012, matching the period of airborne Lidar observations. The study area has significant cloud-free imagery, suggesting the mosaic was closer to the 2012-time frame. The GFC Last data have a spatial resolution of 30 m and four spectral bands, including the Landsat Red and Near Infrared (NIR) bands, as well as 2 Short-wave Infrared (SWIR) bands (Landsat bands 5 and 7). Landsat 7 satellite had a mechanical Scan Line Corrector (SLC) failure in 2003, causing the SLC artifact in the data since then. Although there have been quite a few approaches to fill the SLC gaps [40, 41], the inconsistency still exists particularly in the 1st and 4th bands of GFC Last data. In our study area, SLC effect is not quite visible due to the filling of Landsat 5 data. We also expect the use of other satellite products can compensate the erroneous representation of land surface due to the SLC artifact.

The second input was the digital elevation model (DEM) data derived from the Shuttle Radar Topography Mission (SRTM). SRTM data were acquired from a single-pass interferometry SAR system on board the Space Shuttle Endeavour during the 11-day mission in 2000 [42]. National Aeronautics and Space Administration (NASA) released a new version of void-filled SRTM elevation data (known as “SRTM v3”) in 2014 at a spatial resolution of 1 arc second (approximately 30 m), which is the original resolution of the C-band radar with global coverage. The SRTM v3 data have been void-filled with elevation data primarily from ASTER GDEM (Global Digital Elevation Model Version 2). For tropical forests, SRTM v3 data may not well represent the surface of the ground, as shorter wavelengths such as C-band reflect mainly from top canopies [43]. This enables the estimates of vegetation canopy height if the elevation of bare ground is known [44]. On the other hand, even if the ground is unknown, this topographic information is valuable and highly correlated with forest structure [45, 46].

The third satellite observation used in the analysis was from the Advanced Land Observing Satellite “DAICHI” (ALOS) radar backscatter. The Phased Array L-band Synthetic Aperture Radar (PALSAR) sensor aboard ALOS is an L-band SAR scanner at the wavelength of 1270 MHz enables all-weather land observations without the influence of atmosphere. Japan Aerospace Exploration Agency, JAXA, has produced the new 4 year-25 m spacing global PALSAR mosaics collected globally from 2007 to 2010 using the accurate SAR processing [47]. SAR backscatter data is slope corrected and orthorectified using the SRTM v3, and the radiometrically calibrated [48, 49]. The radiometric calibration had no major artifacts over the study area because of the relatively flat variations of the elevation. We used both HH and HV polarization data in our study, expecting that the deeper penetration of L-band SAR can capture more information on the internal structure of forests.

### Data preprocessing

MCH is the variable of interest in this study. We aggregated the Lidar-derived 1-m CHM product into 100-m resolution using spatial averaging for further analysis. The maps were further filtered using the 1-ha land cover map developed for the Mouila palm oil plantation project from Landsat imagery [33], and only vegetated pixels with MCH higher than 1 m were kept for our analyses. From the study region covering a total of 40 thousand ha, we removed 4.7 thousand ha non-vegetated area out of our study based on land cover map, and an additional 600 ha using the MCH filter. Remote sensing data are the necessary inputs in our study to predict the MCH. We used all four bands of GFC Last, two bands (HH/HV) of ALOS PALSAR, and SRTM v3 data. We also included the local standard deviation of SRTM as an additional input layer, to capture the local variation of topography as an indicator of the variations of canopy structure within a pixel [15, 46].

To account for texture information due to spatial autocorrelation, we created texture layers using Gaussian filters:

1 $$W_{g} \left( {x,y} \right) = \exp \left( { - \frac{{x^{2} + y^{2} }}{{2\sigma^{2} }}} \right)$$

where *W*_*g*_ is the weight assigned to the pixel, *x* and *y* are the cardinal coordinates relative to the pixel of interest (central pixel) located at the origin, and *σ* is the standard deviation of the Gaussian distribution. The result of image filter is the weighted sum (convolution) of neighboring input pixels,

2 $$I = \mathop \sum \limits_{x} \mathop \sum \limits_{y} W\left( {x,y} \right)z\left( {x,y} \right)$$

where $W\left( {x,y} \right)$ is the normalized weight of *W*_*g*_ at each point (*x*, *y*). We created four Gaussian texture layers at different spatial scale using neighborhood windows of 5 × 5, 9 × 9, 17 × 17 and 33 × 33, with the parameter *σ* assigned as 1, 2, 4 and 8 respectively. In addition, we used the measure of the spatial variation by calculating the local standard deviation as an extra texture layer:

3 $$S = \sqrt {\frac{1}{N}\mathop \sum \limits_{x} \mathop \sum \limits_{y} \left( {z\left( {x,y} \right) - \mu } \right)^{2} }$$

where *μ* is the spatial mean of the neighborhood window. For local standard deviations, we selected the same window sizes as the Gaussian filters. We generated these multi-scale texture layers for each satellite input bands from Landsat, ALOS and SRTM.

### Machine learning algorithms

Supervised learning is an approach of building a statistical model from known values of predictor variables (marked as *X*) and the response variable (in our case, *MCH*) for several well studied locations, and to predict the unknown *MCH* for locations where *X* is available. We used two supervised machine learning algorithms that were proved successful in the past ecological studies to perform our study of MCH mapping. Besides preparing the input layers carefully for training and prediction, we also adjusted the models to better suit our needs. Cross validation using existing data is the common practice in machine learning to find the best parameters and avoid overfitting. In this study, we used Monte Carlo cross validation (CV), i.e., the repeated random subsampling [50, 51] to find the best model parameters and uncertainty analyses. All the training samples were randomly selected over the entire scenes. The sample size was either fixed at 400 to study algorithm performance (e.g. Figs 2, 3, 4; Table 1), or changed exponentially to test the sample size effect (Fig. 5).

#### Maximum entropy (ME)

Maximum entropy is a probability-based algorithm that seeks the probability distribution by maximizing the information contained in the existing measurements [52, 53]. The method is used as a classification approach and each class has some probability of occurrence $p\left( {A_{k} } \right)$, where *A* is a measurement event of the response variable, while the measurements are from training samples that belong to class *k*. We have the following constraint that probabilities of all $p\left( {A_{k} } \right)$ must sum to 1.

4 $$\mathop \sum \limits_{k} p\left( {A_{k} } \right) = 1$$

From information theory, the most uncertain probability distribution is the one that maximizes the entropy term:

5 $$E = - \mathop \sum \limits_{k} p\left( {A_{k} } \right)\ln p(A_{k} )$$

This process will ensure that the distribution is estimated by keeping the randomness of samples for the largest entropy. Equation (5) naturally gives the maximum value for the entropy when all probabilities are equal (randomness) assuming no other constraints applied to the system except for the Eq. (4). If we have additional information, i.e., some known MCH observations and corresponding measurements in *X*—we refer to these as the training set, the probability distributions are “conditioned” on the available observations:

6 $$p\left( {A_{k} |\varvec{X}} \right) = p_{k} \left( \varvec{X} \right)p_{0} \left( {A_{k} } \right)/p\left( \varvec{X} \right)$$

The right part of the above equation follows the Bayes’ theorem, meaning that the posterior probability $p\left( {A_{k} |\varvec{X}} \right)$ depends on the distribution of *X* and equals to the product of prior probability $p_{0} \left( {A_{k} } \right)$ and the probability distribution $p_{k} \left( \varvec{X} \right)$ that finds *X* to be in the class *k*, and normalized by the probability distribution of *X* for the entire domain of measurement variables (here satellite images). The maximization of the entropy term in Eq. (5) is equivalent to finding the probability distribution $p_{k} \left( \varvec{X} \right)$ closest to $p\left( \varvec{X} \right)$, and the maximum entropy procedure gives us the “raw” output: $p_{k}^{raw} \left( \varvec{X} \right) = p_{k} \left( \varvec{X} \right)/p\left( \varvec{X} \right)$ [54]. The prior probability $p_{0} \left( {A_{k} } \right)$ is often unknown, as this quantity is the proportion of all observations over the entire scene that belongs to class *k*. Assuming that the training set is sampled randomly, we can estimate $p_{0} \left( {A_{k} } \right)$ as $p_{0} \left( {A_{k} } \right) = N_{k} /N_{total}$, where *N*_*k*_ is the number of samples in the training set labeled as class *k*, and *N*_*total*_ is the total number of samples in the training set.

For our interested metric MCH, we can categorize the numeric values into a set of classes: $k_{1} , k_{2} , k_{3} , \ldots , k_{n}$, where $0 < k_{1} \le MCH_{1} < k_{2} \le MCH_{2} < \ldots < k_{n} \le MCH_{\hbox{max} }$. And each class has a nominal value of MCH—usually the mean value of each class, $\overline{MCH}_{k}$. To predict the MCH value for any pixel *i* with known measurements *X*_*i*_, we calculate it as the expectation of all classes given the ME results retrieved from the training set:

7 $$\left\langle {MCH_{i} } \right\rangle = \frac{{\mathop \sum \nolimits_{k = 1}^{N} p\left( {A_{k} |X_{i} } \right)\overline{MCH}_{k} }}{{\mathop \sum \nolimits_{k = 1}^{N} p\left( {A_{k} |X_{i} } \right)}} = \frac{{\mathop \sum \nolimits_{k = 1}^{N} p_{k}^{raw} \left( {X_{i} } \right)p_{0} \left( {A_{k} } \right)\overline{MCH}_{k} }}{{\mathop \sum \nolimits_{k = 1}^{N} p_{k}^{raw} \left( {X_{i} } \right)p_{0} \left( {A_{k} } \right)}}$$

Empirical tests have found that the model performs better by assigning higher weights to more probable classes. Therefore, we assign a power function to the “raw” output in Eq. (7),

8 $$\left\langle {MCH_{i} } \right\rangle = \frac{{\mathop \sum \nolimits_{k = 1}^{N} \left[ {p_{k}^{raw} \left( {X_{i} } \right)} \right]^{m} p_{0} \left( {A_{k} } \right)\overline{MCH}_{k} }}{{\mathop \sum \nolimits_{k = 1}^{N} \left[ {p_{k}^{raw} \left( {X_{i} } \right)} \right]^{m} p_{0} \left( {A_{k} } \right)}}$$

In our practice, *m* = 3 has been found to be the best parameter with the smallest average relative error and keeping most test points aligned with the 1-to-1 line [15, 34]—we denote this model as the MEBC—the bias-corrected ME model.

#### Random forests (RF)

Random forests (RF) algorithm is an ensemble model of decision trees trained from randomly selected subset features and random sampling of the training set using bagging method [36]. RF can be a regression method when using regression trees, and for the *j*th regression tree, the regression model can be built as

9 $$MCH = f_{j} \left( x \right) + \varepsilon$$

where $x \in \varvec{X}$ is the bagged samples of the training set, $f_{j} \left( \cdot \right)$ is the non-parametric function determined by the *j*th regression tree. The final prediction of RF regression is the unweighted average of the collection of trees:

10 $$\widehat{MCH}(\varvec{X}) = \frac{1}{J}\sum\limits_{j = 1}^{J} {f_{j} (x)}$$

This averaging process inevitably creates results biased towards the sample mean, and large/small values of MCH are often underestimated/overestimated. Various bias correction methods have been proposed to post RF results [55–57]. In our study, we modified the bootstrap bias correction method [55] and implemented a second RF run to correct the biases. Acknowledging that there is a systematic bias signal in the original RF, the new response variable for the second RF can be defined as the out-of-bag estimation of MCH minus the regression residual,

11 $$MCH_{new} = \widehat{MCH}_{oob} \left( \varvec{X} \right) - \left( {MCH - \widehat{MCH}_{oob} \left( \varvec{X} \right)} \right) = 2\widehat{MCH}_{oob} \left( \varvec{X} \right) - MCH$$

where $\widehat{MCH}_{oob} \left( \varvec{X} \right)$ is the out-of-bag estimation of MCH for the training data, and the difference between $\widehat{MCH}_{oob} \left( \varvec{X} \right)$ and original MCH is the regression residual from the original RF. Our second RF run tries to capture the systematic regression bias of original RF by estimating the new metric (*MCH*_*new*_) that is further biased toward the opposite direction of the original MCH. Therefore, we obtain the new RF model $\left( {\widehat{MCH}_{new} (X) = \frac{1}{J}\mathop \sum \limits_{j = 1}^{J} f'_{j} \left( x \right)} \right)$. For a new set of samples $x^{o} \in \varvec{X}^{\varvec{o}}$, the bias-corrected RF prediction ($\widehat{MCH}_{BC} \left( {X^{o} } \right)$) can be written as

12 $$\widehat{MCH}_{BC} \left( {X^{o} } \right) = \widehat{MCH}\left( {\varvec{X}^{\varvec{o}} } \right) - \left( {\widehat{MCH}_{new} \left( {\varvec{X}^{\varvec{o}} } \right) - \widehat{MCH}\left( {\varvec{X}^{\varvec{o}} } \right)} \right) = 2\widehat{MCH}\left( {\varvec{X}^{\varvec{o}} } \right) - \widehat{MCH}_{new} (\varvec{X}^{\varvec{o}} )$$

We denote the bias-corrected RF as RFBC model in our study.

### Statistical assessments

To evaluate the performance of the spatial modeling algorithms, we used three statistical measures to evaluate the CV test results: the coefficient of determination (R^2^), the root-mean-square error (RMSE), and the mean signed deviation (MSD). We applied all these measures to an independent test set, where the original MCH is obtained from airborne Lidar, while the predicted MCH is derived using the satellite inputs and the model trained from the training set. Besides the overall MSD over all test samples, we assessed two additional MSD measures for both small trees (MSD1) and large trees (MSD2). We define MSD1 as the MSD calculated for test samples with the sum of predicted MCH and measured MCH to be less than 20 m. Similarly, MSD2 is defined as MSD for samples with the sum of predicted MCH and measured MCH to be more than 60 m. Also, we calculated the semi-variograms [58] for original MCH as well as the model residuals to quantify the spatial autocorrelation in the data.

### Data simulation

We generated simulation data to explain the prediction biases of RF and RFBC. The data generation process is (1) creating five normalized random variables of X with zero mean and one standard deviation, and 20 % correlation between each other, (2) generating Y to be 10 times of the mean value of Xs plus a constant 20, with 10 % perturbation following Gaussian distribution, and (3) adding noise on Xs. The noise on Xs has two scenarios: (a) random Gaussian noise with 20 % perturbation over the original X distribution (Blue curves of sample distribution in Fig. 7), and (b) random noise with 80 % perturbation over the original X distribution (Red curves of sample distribution in Fig. 7). The second scenario was used to simulate the hypothesis that satellite data cannot capture the full range of variance of forest structure due to the insensitivity of satellite measurements to vertical vegetation structure. Original RF and RFBC were performed on this simulated data with half of the data as training and the rest as the independent test set.

## Abbreviations

AGB: aboveground biomass
ALOS: advanced land observing satellite “DAICHI”
CHM: canopy height model
CV: cross validation
DEM: digital elevation model
DSM: digital surface model
DTM: digital terrain model
GDEM: global digital elevation model version 2
GFC: global forest change
GHG: greenhouse gas
JAXA: Japan Aerospace Exploration Agency
MCH: mean canopy height
ME: maximum entropy
MEBC: bias-corrected maximum entropy
MSD: mean signed deviation
MSD1: mean signed deviation for small trees
MSD2: mean signed deviation for large trees
NASA: National Aeronautics and Space Administration
NIR: near infrared
PALSAR: phased array L-band synthetic aperture radar
REDD: reduced emissions from deforestation and degradation
RF: random forest
RFBC: bias-corrected random forest
RMSE: root-mean-square error
SAR: synthetic aperture radar
SLC: scan line corrector
SRTM: shuttle radar topography mission
SWIR: shortwave infrared
